# Potential regulatory role of the Nrf2/HMGB1/TLR4/NF-κB signaling pathway in lupus nephritis

**DOI:** 10.1186/s12969-023-00909-5

**Published:** 2023-10-23

**Authors:** Shi-jie Li, Dan-dan Ruan, Wei-zhen Wu, Min Wu, Qiu-yan Wu, Han-lu Wang, Yuan-yuan Ji, Yan-ping Zhang, Xin-fu Lin, Zhu-ting Fang, Li-sheng Liao, Jie-wei Luo, Mei-zhu Gao, Jia-bin Wu

**Affiliations:** 1 Fujian Provincial Hospital, Shengli Clinical Medical College of Fujian Medical University, Fuzhou, 350001 China; 2 Xiyuan Clinical Medical College of Beijing University of Chinese Medicine, Beijing, 100029 China; 3 Department of Hematology, Fujian Provincial Hospital, Fuzhou, 350001 China; 4 Department of Traditional Chinese Medicine, Fujian Provincial Hospital, Fuzhou, 350001 China; 5 Department of Nephrology, Fujian Provincial Hospital, Fuzhou, 350001 China; 6 School of Medicine, Fuzhou Second Hospital, Xiamen University, Fuzhou, 350007 China; 7 The Third Clinical Medical College, Fujian Medical University, Fuzhou, 350007 China

**Keywords:** Autoimmune disease, HMGB1, LN, Nrf2, TLR4 ^+^ CXCR4 ^+^ plasma cells

## Abstract

**Objectives:**

Systemic lupus erythematosus is an autoimmune disease that involves multiple organ systems. One of its major complications, lupus nephritis (LN), is associated with a high mortality rate, and children-onset LN have a more severe course and worse prognosis than adults. Oxidative stress and inflammatory responses are involved in LN development and pathogenesis. Thus, this study aimed to explore the role of signaling regulation of the Nrf2/HMGB1/TLR/NF-κB pathway in LN pathogenesis and unravel the expression of TLR4^+^CXCR4^+^ plasma cells subset (PCs) in LN.

**Methods:**

C57BL/6 and MRL/lpr mice were divided into four groups: control, model, vector control, and Nrf2 overexpression groups. The vector control and Nrf2 overexpression groups were injected with adenoviral vectors into the kidney in situ. Pathological changes in kidney tissues were observed by hematoxylin–eosin staining. The expression of Nrf2, HMGB1, TLR4, NF-κB, and downstream inflammatory factors in kidney samples was analyzed by quantitative polymerase chain reaction, western blotting, and enzyme-linked immunosorbent assay. The ratios of TLR4^+^CXCR4^+^ PC subsets in the blood and kidneys of mice were determined by flow cytometry.

**Results:**

In MRL/lpr mice, Nrf2 was downregulated while HMGB1/TLR4/NF-κB pathway proteins were upregulated. Nrf2 overexpression decreased the expression of HMGB1, TLR4, NF-κB, and its downstream inflammatory cytokines (IL-1β and TNFα). These cytokines were negatively correlated with an increase in Nrf2 content. PC and TLR4 ^+^ CXCR4 ^+^ PCs in the blood and kidney samples were significantly increased in MRL/lpr mice; however, they were decreased upon Nrf2 overexpression.

**Conclusion:**

This study showed severe kidney injury in an LN mouse model and an increased ratio of TLR4^ +^ CXCR4 ^+^ PCs. Furthermore, we observed that Nrf2 regulates LN immune response through the Nrf2/HMGB1/TLR4/NF-κB pathway, which can be considered an important target for LN treatment. The clinical value of the findings of our study requires further investigation.

## Introduction

Systemic lupus erythematosus (SLE) is an autoimmune disease involving multiple systems, characterised by the production of autoimmune antibodies and the deposition of immune complexes that cause damage to a number of organs [1–3]. The prevalence of SLE worldwide is about 43.7 (15.87 to 108.92) per 100,000 persons, with large variations by sex, age group and geographical region [4]. Approximately 15–20% of SLE patients begin in childhood, with a more severe course and worse prognosis than adult SLE. LN occurs in up to 80 per cent of childhood-onset SLE (cSLE), contributing to the high mortality rate [5, 6]. At present, most of the treatment of childhood-onset LN (cLN) is inferred from adults, so it is particularly important to delve into the pathogenesis of LN and discover potential therapeutic targets. There are many similarities in the pathophysiological mechanisms of adult and cSLE, and it is generally accepted that the pathogenesis is due to the deposition of immune complexes formed by apoptotic cell-derived chromatin and anti-nuclear antibodies in the kidney, inducing cytokine secretion imbalance and lymphocyte dysfunction and resulting in an improper immune response[7–9]. Furthermore, in patients with SLE, macrophages show a decreased ability to clear apoptotic fragments, resulting in prolonged exposure of the immune system to self-antigens [10, 11]. Nuclear factor erythroid-derived 2-related factor 2 (Nrf2) is key to defense against oxidative stress [12, 13]. Nrf2 has a wide range of regulatory actions; however, it acts mainly through the Kelch-like ECH-associated protein (Keap1)-dependent pathway in which its transfer to the nucleus occurs, activating the downstream antioxidant response element (ARE) and exerting its antioxidant effect [3, 14].

High mobility group protein box1 (HMGB1) is a typical damage-associated molecular pattern (DAMP) molecule associated with various disease phenotypes, including SLE [15]. As the main substance produced by apoptosis, HMGB1 can bind self-antigen components such as nucleosomes. Subsequently, through receptors for advanced glycation end products (RAGE), toll-like receptor 2 (TLR2), and toll-like receptor 4 (TLR4), the release of inflammatory factors [such as interferon (INF) and nuclear factor kappa B (NF-κB)] is promoted, along with autoantibody production of mainly anti-nuclear antibodies through immune system activation [15]. The inflammation-mediated role of the HMGB1/TLR4/NF-κB signaling pathway in diseases such as asthma, rheumatoid arthritis, lung injury, sepsis, and SLE and its associated complications (such as LN) has received widespread attention. Furthermore, therapies targeting this signaling pathway have achieved preliminary success [16–21].

Toll-like receptors (TLRs) are type I transmembrane proteins and pattern recognition receptors that recognize various pathogen-associated molecular patterns and DAMPs [22]. After TLR4 recognition by extracellular HMGB1, AP-1 and IRF3/5/7 are upregulated and promote the transfer of NF-κB to regulate the expression of genes encoding inflammatory cytokines through the TLR4 adapter MyD88 and TRIF [23–25].TLR4 is involved in autoimmunity and LN, as well as NF-κB activity is essential for immune cell activation [26–28]. Compared with C57BL/6(lpr/lpr) mice, TLR2- and TLR4-deficient mice show lower anti-nuclear and anti-phospholipid autoantibody levels, less kidney damage, and milder disease [29].

In abnormal autoimmune tolerance, autoreactive B cells (i.e., pathogenic plasma cells) produce large amounts of mainly anti-nuclear and anti-DNA autoantibodies [30]. Plasma cells (PC) differentiate from the germinal centers of peripheral lymph nodes to become memory B cells or long-lived PCs [30]. Long-lived PCs determine the permanent production of autoantibodies. Several studies have explored the role of TLR signaling in B cells in LN. TLR7 is deleterious during SLE development, whereas TLR9 confers protection. TLR7-deficient lupus mice show autoimmune inflammation remission, whereas TLR9-deficient lupus mice show a more severe autoimmune inflammation [31, 32]. Intrinsic TLR4 signal transduction in B cells plays an important role in autoimmune diseases, which differs from the role in the HMGB1/TLR4/NF-κB inflammatory signaling pathway. C57BL/6 (lpr/lpr)-TLR4 deficient mice have a reduced number of marginal-zone-B cells caused by decreased expression of B lymphocyte stimulator receptors, which affects B cell maturation [29]. TLR4 may also enhance autoantibody production through the increased expression of Th1- and Th17-related cytokines [28]. Chemokines, particularly CXCR4 and CXCL12, are strongly associated with B-cell infiltration in the kidneys of lupus-prone MRL/lpr mice [33]. TLR4^+^CXCR4^+^ PCs are long-lived PCs that are positively correlated with anti-dsDNA levels in the serum and kidneys, leading to autoantibody production and LN development [34]. However, there are few studies on B cell TLR4 signaling in autoimmunity. The correlation between TLR4^+^CXCR4^+^ PCs, as one of the characteristics of autoreactive B cell subsets, and LN development requires further exploration.

## Materials and methods

### Animal models

Six C57BL/6 mice (female, 60–80 g, specific-pathogen-free (SPF) grade, 11 weeks old) and 18 lupus erythematosus MRL/lpr mice (female, 61–82 g, SPF grade, 11 weeks old) were purchased from Huachuang sino Pharmaceutical Technology Co., LTD. (Taizhou, Jiangsu, China).

### Experimental design

This study was approved by the Experimental Animal Welfare and Ethics Committee of ZVAST BIO Co., LTD. (No.2021100701). All six C57BL/6 mice and the 18 MRL/lpr mice were randomly divided into each group. The experiment was carried out in two parts:

1. Experimental protocol A: for HE staining and flow cytometry. It was divided into the following four groups: control group, MRL/lpr group (hereafter referred to as the model group), MRL/lpr mice with Nrf2 gene adenovirus overexpression interference empty-load group (hereafter referred to as the Nrf2 empty-load group), and MRL/lpr mice with Nrf2 gene adenovirus overexpression interference vector group (hereafter referred to as the Nrf2 vector group). Three mice were randomly assigned to each group(*n* = 3), with C57BL/6 mice in the control group and MRL/lpr mice in another three groups.
2. Experimental protocol B: for genetic testing in mice, including qPCR, Western Blotting, ELISA detection. It was also divided into four groups: control group, model group, Nrf2 empty-load group, Nrf2 vector group. Three mice were randomly assigned to each group (*n* = 3), with C57BL/6 mice in the control group and MRL/lpr mice in another three groups.

All animals entered the experiment after 1 week of adaptive feeding. Throughout the experiment, animals were fed a standard diet and had ad libitum access to food and water. Animals were maintained in a clean room at a temperature of 20–26 °C and humidity of 40–70%. The levels of circulating immune complexes in MRL/lpr mice increased significantly at approximately 12 weeks after birth. Therefore, 12-week-old mice were chosen as the starting point for this study. The experiments lasted for four weeks, starting at the beginning of the 12^th^ week of age and ending by the 16^th^.

Adenoviral plasmids containing the Nrf2 gene (pAdEasy-EF1-MCS-CMV-EGFP-Nrf2, the same sequence as NM_010902.4) and an empty vector plasmid (pAdEasy-EF1-MCS-CMV-EGFP) were purchased from Hanbio BIO Co., Ltd. (Shanghai, China). Mice in the Nrf2 empty-load and Nrf2 vector groups were anesthetized, the abdominal skin was pre-prepared, and the renal pelvis was bilaterally exposed. Each mouse was injected bilaterally into the renal pelvis with 50 μl of virus, and the skin was subsequently sutured. Overexpression of Nrf2 was verified by immunofluorescence before proceeding with additional experiments.

On the one week after orthotopic virus injection, the kidneys of mice in each group were collected, embedded in paraffin, sectioned, and stained for renal histology by hematoxylin–eosin (HE) staining. Enzyme-linked immunosorbent assay (ELISA) was performed in mouse serum to detect the levels of IL-1β, TNF-α, and NF-κB. Quantitative polymerase chain reaction (qPCR) was performed to detect Nrf2 (Nfe2l2, NM_010902.4), HMGB1 (NM_ 010439.4), and TLR4 (NM_021297.3) expression in kidney tissues. Western blotting (WB) was conducted to evaluate Nrf2, HMGB1, and TLR4 protein levels in kidney tissues. Flow cytometry was employed to detect the TLR4^+^CXCR4^+^ PC ratio in the blood and kidney.

### HE staining

The mouse kidney tissue samples were dehydrated, soaked, embedded in wax, and sectioned using a microtome. Samples were then stained with hematoxylin and eosin (HE) according to the manufacturer’s guidelines, dehydrated recursively with a series of ethanol dilutions, and sealed with xylene. Images were acquired using a microscope.

### Flow cytometry

A single-cell suspension of approximately 10^7^ cells/ml was prepared, and 100 μl was added to a flow tube. Five µL of each reagent (CD38 FITC, CD138 PE, CD27 APC)/(CXCR4 APC, TLR4 PE) were added to the cell suspension, incubated in the dark for 30 min, and centrifuged at 400 × *g* for 5 min at room temperature. The supernatant was discarded, 2 mL of PBS was added, and the sample was centrifuged again at 400 × *g* for 5 min at room temperature, with a subsequent repetition of these processes. After centrifugation, 500 µL of PBS and a flow cytometer probe (NovoCyte 2060R, ACEA BIO Co., Hangzhou, China) were added for detection. CD38 FITC, CD138 PE, CD27 APC, CXCR4 APC, and TLR4 PE were purchased from BioLegend (San Diego, CA, USA).

### ELISA detection

Blood samples were collected and centrifuged for serum retrieval. All samples and reagents from the IL-1β (MM-0040M1, MEIMIAN, Jiangsu, China), TNF-α (MM-0132M1, MEIMIAN, Jiangsu, China), and NF-κB kits (ML063331-2, MEILIAN, Shanghai, China) were equilibrated at room temperature for 120 min before starting the protocol. The ELISAs were conducted according to the manufacturer’s instructions. Briefly, 100 μL of horseradish peroxidase (HRP)-labeled detection antibody were added to the samples in the well and incubated for 60 min in a closed thermostat. After plate washing five times, 50 μL each of substrate A and B were added to each well and incubated in the dark at 37 °C for 15 min. Then, 50 μL of stop solution were added to each well, and the optical density value was measured at a wavelength of 450 nm using a microplate reader (WD-2012B, LIUYI BIO Co., Ltd., Beijing, China).

### qPCR

Kidney tissue samples were processed using the TRIzol Reagent kit (CW0580S, CWBIO, Beijing, China), total RNA was extracted using an Ultrapure RNA Extraction Kit (CW0581M, CWBIO, Beijing, China), and RNA concentration and purity were determined using an ultraviolet spectrophotometer (NP80, Implen NanoPhotometer, Munich, Germany). RNA was converted into cDNA using HiScript II Q RT SuperMix for qPCR (+ gDNA wiper) (R223-01, Vazyme, Nanjing, China) and used for fluorescent quantitative PCR, with the following reaction parameters: 10 μl of 2 × SYBR Green PCR Master Mix (Q711-02, Vazyme, Nanjing, China), 1 μl of cDNA, 0.4 μl of forward primers, 0.4 μl of reverse primers, and 8.2 μl of RNase-free ddH_2_O. The qPCR program consisted of 40 cycles of amplification. β-actin was used as the housekeeping gene, and the relative expression of Nrf2, HMGB1, TLR4, and NF-κB was calculated according to the 2^−△△Ct^ method. (Primer sequences are shown in Table 1).

**Table 1:** Primers used for qPCR experiments

Primer name	Primer sequence	Primer length (nt)	Product length (bp)	Annealing temperature (℃)
Nrf2 F	CTTTAGTCAGCGACAGAAGGAC	22	227	47.8
Nrf2 R	AGGCATCTTGTTTGGGAATGTG	22		
HMGB1 F	GGCGAGCATCCTGGCTTATC	20	86	60.1
HMGB1 R	GGCTGCTTGTCATCTGCTG	19		
TLR4 F	GAAGCTTGAATCCCTGCATAGAGGT	25	249	49.0
TLR4 R	AGTTTGAGAGGTGGTGTAAGCC	22		
NF-κB F	CACGAGGCTCCTTTTCTCAA	20	270	58.3
NF-κB R	GGGGTTCAGTTGGTCCATTG	20		
β-actin F	AGGGAAATCGTGCGTGAC	18	192	58.0
β-actin R	CATACCCAAGAAGGAAGGCT	20		

### Western blotting

Mouse kidney samples were treated with radioimmunoprecipitation assay (RIPA) buffer for protein extraction. Protein concentration was subsequently determined using a BCA kit. The samples were denatured for Western blotting. Briefly, protein samples were loaded into the gel, and sodium dodecyl benzene sulfonate gel electrophoresis was performed for 1–2 h. Proteins were then transferred to the PVDF membrane by the wet method, blocked for 1 h in a 3% TBS-T skim milk solution, and incubated overnight in the primary antibody solution at 4 °C. The next day, the membranes were washed three times with standard TBS-T buffer, incubated for 2 h at room temperature in a secondary antibody solution, washed three times, and incubated with ECL prior to detection in an imaging system. Images were analyzed in the "ImageJ" software. The mouse anti-GAPDH antibody was purchased from TransGen Biotech Co., Ltd. (Beijing, China). HRP-conjugated Goat Anti-Mouse IgG (H + L) and HRP-conjugated Goat Anti-Rabbit IgG (H + L) were purchased from Servicebio Technology Co., Ltd. (Wuhan, China). Mouse Anti-Nrf2 was acquired from Proteintech Group Inc. (Chicago, IL, USA), and Rabbit Anti-HMGB1 and Rabbit Anti-TLR4 antibodies were purchased from Affinity Biosciences Ltd. (Melbourne, Australia).

### Statistical analysis

SPSS19.0 software was used for statistical analysis. Data are presented as mean ± standard deviation (SD). Comparisons between three or more groups were conducted using one-way analysis of variance, and then comparisons between the two groups were conducted using the LSD method. Graphpad 8.0 was used for graph design, and the inspection level was set at α = 0.05. Gray values were analyzed using Image Pro J software.

## Results

### Severe kidney damage in MRL/lpr mice

**Fig. 1:**
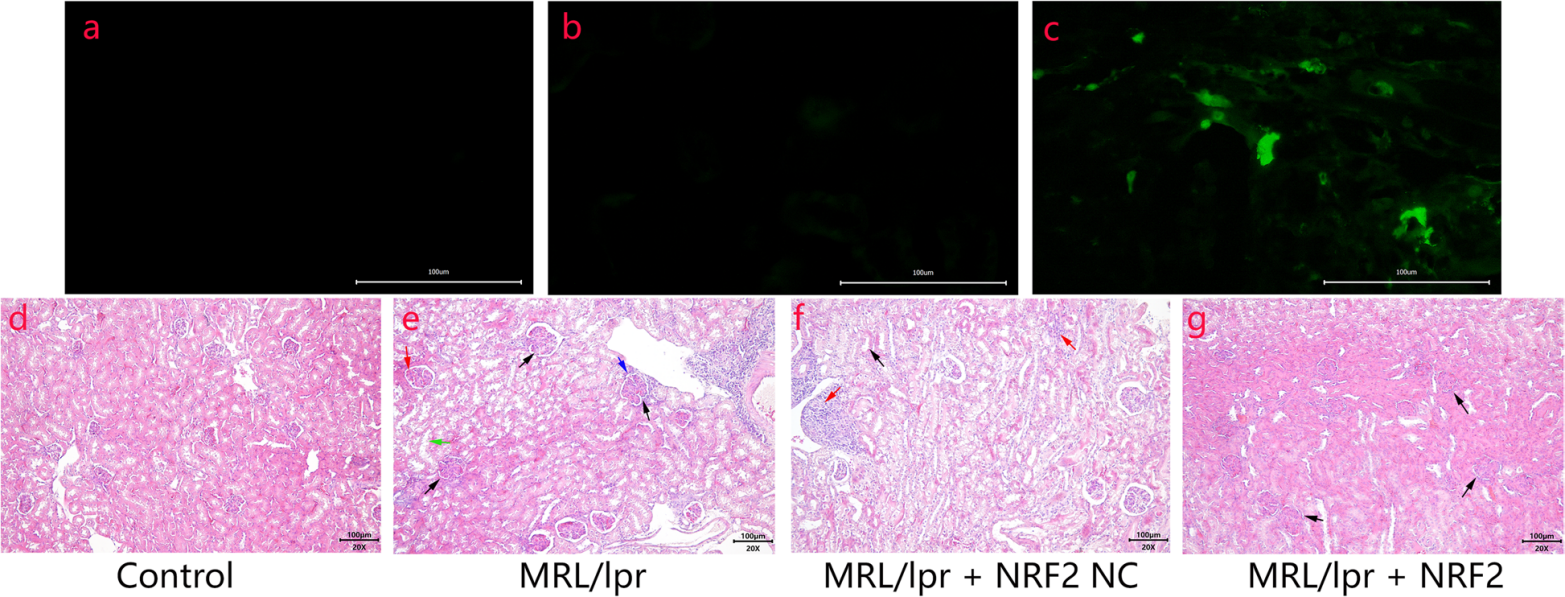
Nrf2 adenovirus verification and HE staining. **a**-**c** Immunofluorescence verification of adenovirus effective transduction: immunofluorescence of Nrf2 vector group (**c**) is significantly stronger than that of control group (**a**) and Nrf2 empty-load group (**b**). **d**-**g** Hematoxylin–eosin staining of kidney histology: in the control group, glomeruli and renal tubules structures are histologically normal with a small amount of hyperemia near some glomeruli but without inflammatory cell infiltration. In MRL/lpr mice (model group), the glomerulus is congested (black arrow) and structurally disordered, a small amount of hyperemia is observed near some glomeruli (red arrow), several renal tubular epithelial cells are exfoliated to the lumen (green arrow), and inflammatory cell infiltration can be detected (blue arrow). In the Nrf2 empty-load group, the renal tubular lumen is congested, the boundary between the cells is unclear (red arrow), several renal tubular epithelial cells shed into the lumen (black arrow), and obvious inflammatory infiltration is observed. In the Nrf2 vector group, the glomerulus is congested (black arrow), and the disorder of tubular structure is alleviated compared with that in the model group

The fluorescence derived from viral infection was significantly stronger in the Nrf2 vector group than in the Nrf2 load-empty and control groups, suggesting the successful construction and transduction of the interference vector (Fig. 1a-c). MRL/lpr mice showed glomerular inflammation and congestion, kidney structural disorder with several renal tubular epithelial cells exfoliated into the lumen, and an obvious infiltration of inflammatory cells. However, this effect was not observed in the control group. In the Nrf2 empty-load group, the tubular lumen was congested, the boundaries between cells were unclear, renal tubular epithelial cells were shed into the lumen, and there was obvious inflammatory infiltration similar to that observed in MRL/lpr mice. The kidneys of mice with Nrf2 overexpression showed slightly congested glomeruli and amelioration of renal tubule structural disorder. Thus, Nrf2 overexpression improved renal tissue injury in MRL/lpr mice (Fig. 1d-g).

### HMGB1/TLR4/NF-κB signaling pathway and downstream inflammatory factors in LN

**Fig. 2:**
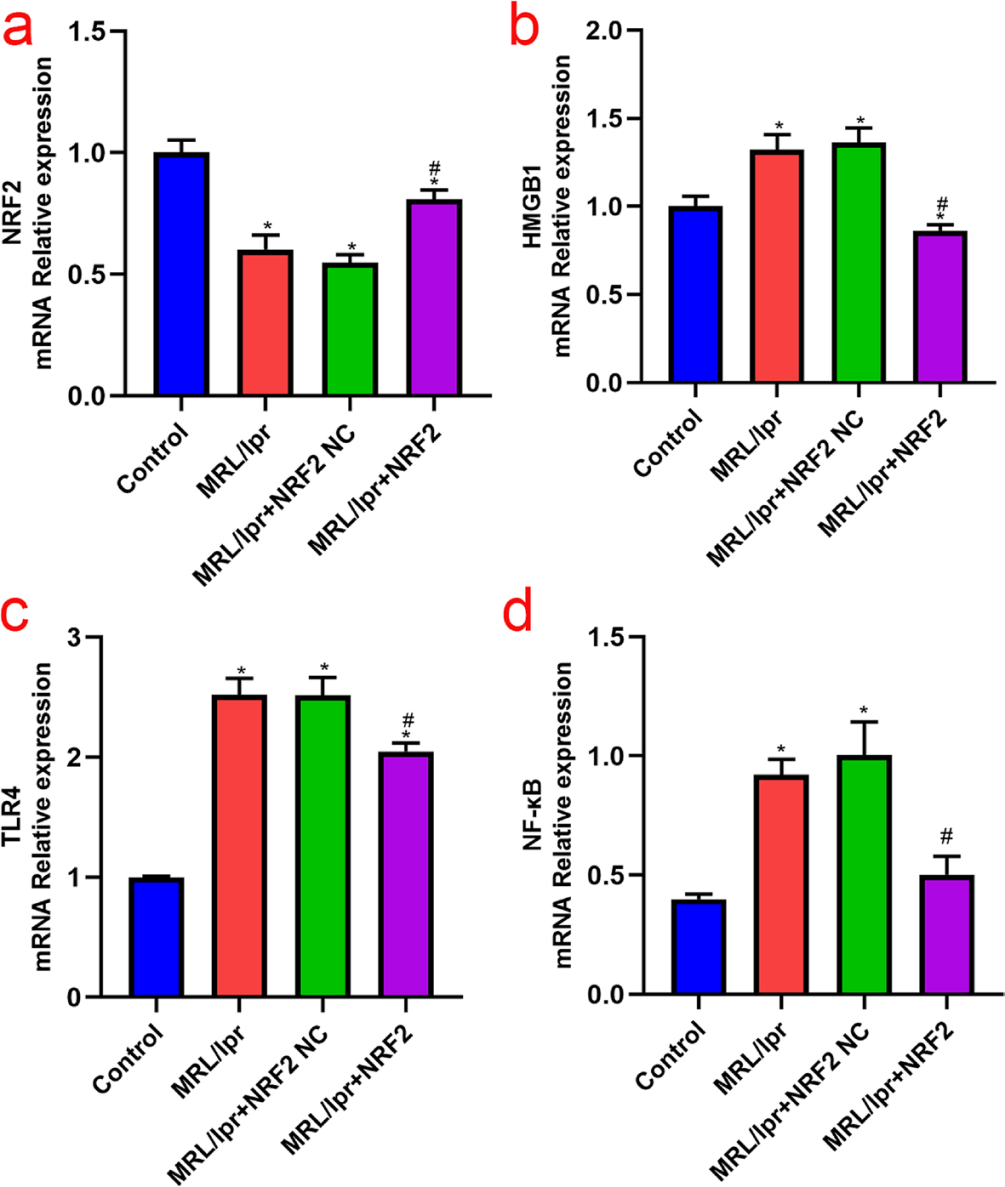
Nrf2, HMGB1, TLR4, and NF- κB mRNA expression in each group, detected by q-PCR. **a**-**d** Nrf2 mRNA expression is significantly decreased and HMGB1, TLR4, and NF-κB expression is significantly increased in the model group compared to that in the control group; in Nrf2 vector group, the expression of Nrf2 mRNA is significantly upregulated, while the expression of HMGB1, TLR4, and NF- κB mRNA is significantly downregulated. (*Compared to the control group, *P* < 0.05; #Compared to the model group, *P* < 0.05, *n* = 3 per group)

**Fig. 3:**
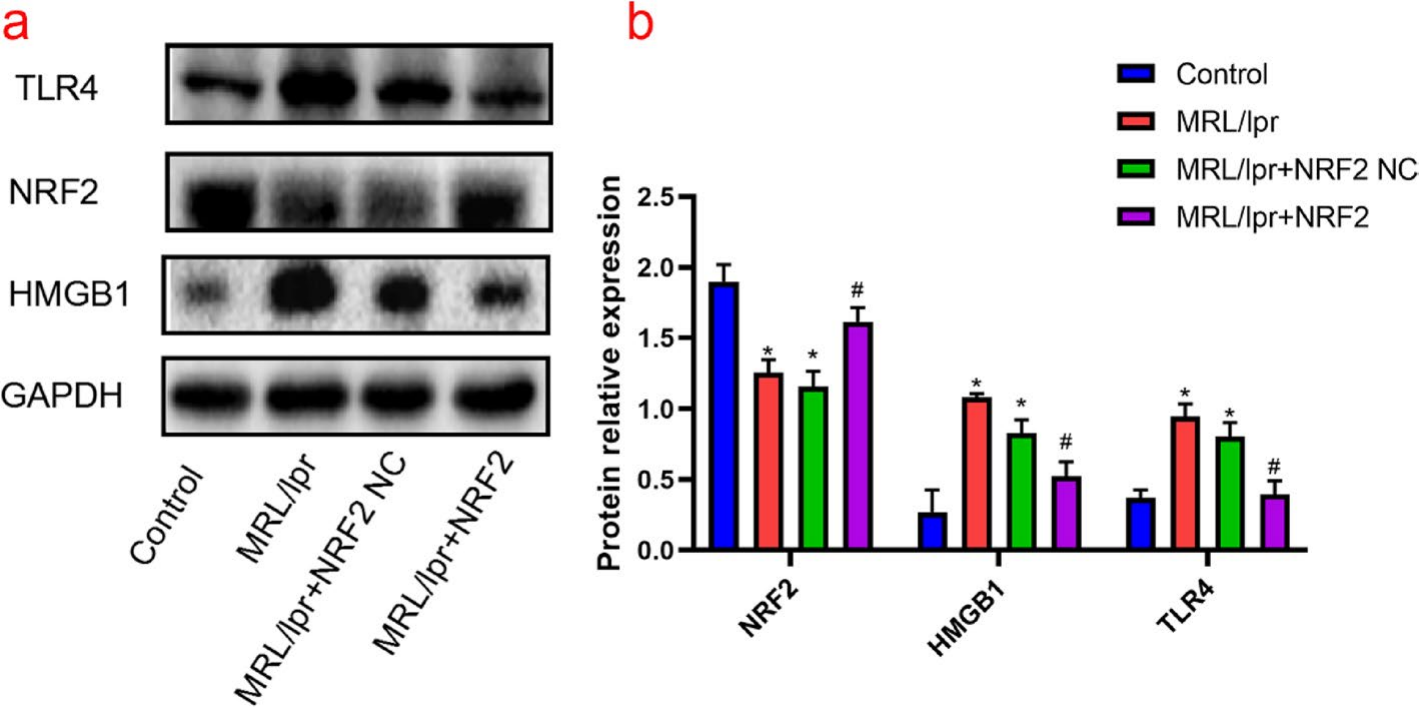
Nrf2, TLR4, and HMGB1 protein levels in each group, detected by western blotting. **a**-**b** Nrf2 protein levels in the model group are decreased, while HMGB1 and TLR4 are increased, compared to that in the control group. Nrf2 overexpression group shows increased Nrf2 protein levels and decreased HMGB1 and TLR4, compared to that in the model group. Nrf2 levels are negatively correlated with those of HMGB1 and TLR4. (*Compared to the control group, *P* < 0.05; #Compared to the model group, *P* < 0.05, *n* = 3 per group)

**Fig. 4:**
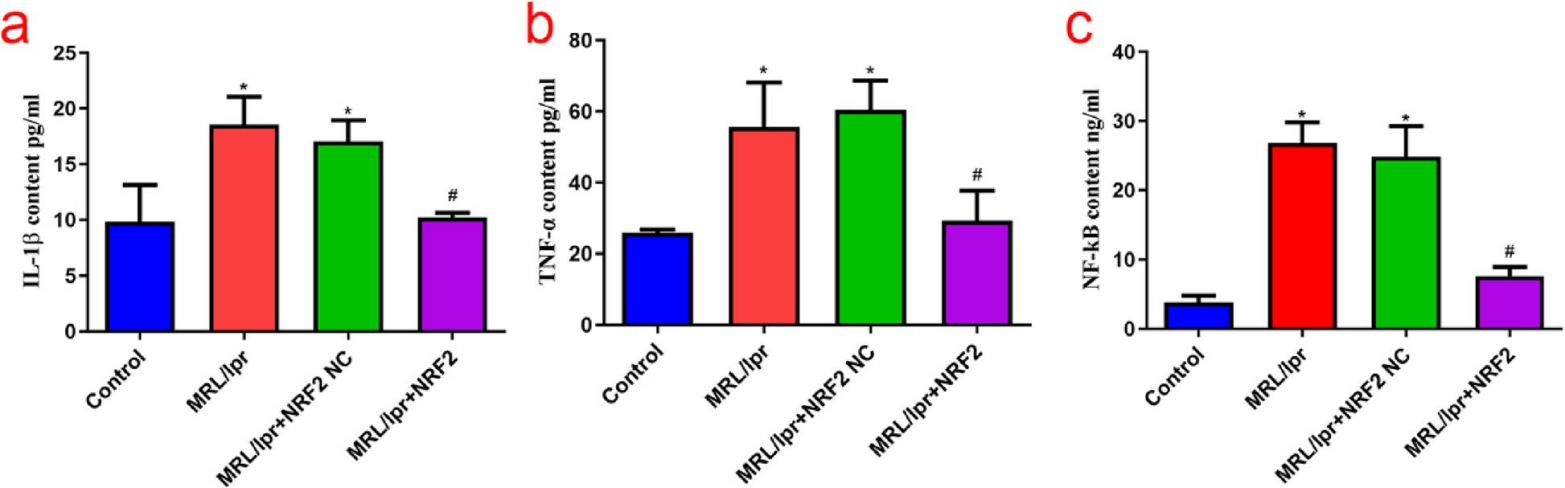
IL-1β, TNF- α, and NF- κB protein levels in each group, detected by ELISA. **a**-**c** The levels of NF- κB, IL-1β, and TNF- α are significantly upregulated in the model group. Nrf2 overexpression promoted a decrease in all these proteins. (*Compared to the control group, *P* < 0.05; #Compared with the model group,* P* < 0.05, n = 6 per group)

The mRNA expression levels of HMGB1, TLR4, and NF-κB in the model group were higher (*P* < 0.05) than those in the control group. However, no significant differences were detected in the mRNA expression of these proteins between the empty load and model groups (Fig. 2). In addition, WB and ELISA showed upregulation of HMGB1, TLR4, NF-κB, and their downstream inflammatory factors (IL-1β and TNFα) in the model group compared to those in the control group (*P* < 0.05) (Figs. 3 and 4). These results suggest that the HMGB1/TLR4/NF-κB signaling pathway is closely related to the occurrence and development of nephritis in a mouse model of lupus.

### Nrf2 regulatory role in the HMGB1/TLR4/NF-κB signaling pathway

The expression of Nrf2 mRNA in the kidneys of MRL/lpr mice was significantly decreased compared to that in the control group, whereas overexpression of Nrf2 increased mRNA expression significantly compared to that in the model group (*P* < 0.05) (Fig. 2). As mentioned in Sect. 3.1, kidney injury in the model group was severe, and Nrf2 overexpression improved glomerular congestion and reduced kidney tissue disorders. Meanwhile, qPCR showed a reduction in HMGB1, TLR4, and NF-κB mRNA levels in the Nrf2 vector group compared with those in the model group (*P* < 0.05). WB and ELISA further supported this decrease, with significantly reduced levels of HMGB1, TLR4, and NF-κB in the Nrf2 vector group, along with their downstream inflammatory factors IL-1β and TNF-α (Figs. 3 and 4).

Altogether, severe kidney injury was observed in the kidney tissue of MRL/lpr mice, resulting in decreased Nrf2 expression. Additionally, the expression levels of HMGB1, TLR4, and NF-κB mRNA and protein increased to different degrees. After Nrf2 overexpression, kidney injury improved, and HMGB1, TLR4, and NF-κB levels were significantly re-established, which showed that Nrf2 and HMGB1/TLR4/NF-κB were negatively correlated in the kidney tissue of MRL/lpr mice.

### TLR4^+^CXCR4^+^ PCs in the blood and kidney tissue of lupus model mice

**Fig. 5:**
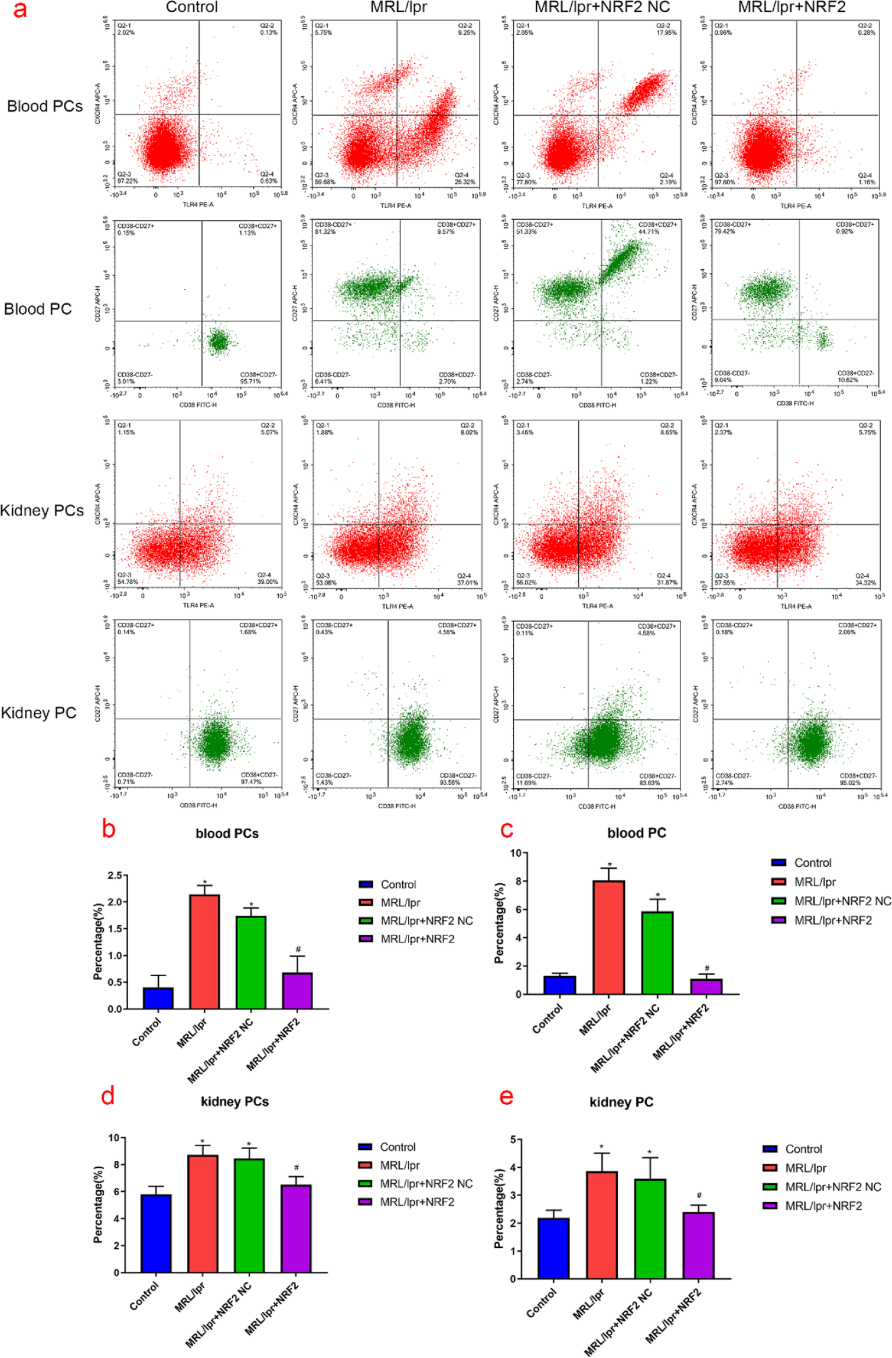
The proportion of plasma cells detected by flow cytometry. **a** Typical representative flow scatter plots for each group: The scatter plot is divided into four sections, the upper right quadrant is double positive cells with the antigen markers indicated by the X-axis and Y-axis respectively, TLR4^+^CXCR4^+^ PCs or CD27^+^CD38^+^ PC, while the lower left quadrant is the opposite, TLR4^−^CXCR4^−^ PCs or CD27^−^CD38.^−^ PC. The upper left and lower right quadrants represent cells that are marked positive only for the Y-axis parameter or the X-axis parameter, respectively. **b** Flow histograms. The proportion of PCs and PC in the kidney and blood of the model group are significantly increased, compared to that of the control group. Nrf2 overexpression lowers this proportion in kindeys and blood than that in the model group.(*Compared with the control group, *P* < 0.05; #Compared with the model group,* P* < 0.05, *n* = 3 per group)

Flow cytometry analysis showed increased the levels of PC and TLR4^+^CXCR4^+^ PCs in the blood and kidney tissue samples of the model group compared to those in the control group (Fig. 5). Nrf2 overexpression decreased both PC and TLR4^+^CXCR4^+^ PCs levels. In contrast, PC and TLR4^+^CXCR4^+^ PCs levels in the blood and kidney of the Nrf2 load-empty group were higher than those of the control group (*P* < 0.05) but showed no significant difference compared to the model group (*P* > 0.05). These results indicate that TLR4^+^CXCR4^+^ PCs were elevated in the blood and kidney tissues of MRL/lpr mice, showing an association with LN.

## Discussion

The absence of the Fas gene in MRL/lpr mice leads to the abnormal clearance of apoptotic debris or necrotic cells. Incompletely degraded nuclear fragments act as self-antigens that activate the innate and adaptive immune system and autoantibody production. The antigen–antibody combination forms a circulating immune complex or an in situ immune complex that can cause kidney tissue damage, triggering an inflammatory response and the production of large amounts of reactive oxygen species (ROS). Subsequently, this activates antioxidant responses and elevates Nrf2. However, with aggravation of inflammation, oxidative stress resistance decreases and LN progression occurs [35, 36]. As an important component of adaptive immunity, B cells differentiate into PCs and secrete autoantibodies. It has been shown that PC differentiation is accompanied by CXCR4 expression induction, and remain positive for this receptor [31, 32]. TLR signaling is indispensable for B lymphocyte homotype transformation and differentiation into secretory PCs, and it is the third signal for the proliferation and differentiation of B lymphocytes [37].

This study showed a significant increase in MRL/lpr mouse blood and kidney PC(Blood PCs 9.25%, Blood PC 9.57%; Kidney PCs 8.02%, Kidney PC 4.55%), which might be involved in the characteristic renal parenchymal damage of LN. TLR4^+^CXCR4^+^ PCs were also significantly higher than those in control mice(Blood PCs 0.13%, Blood PC 1.13%; Kidney PCs 5.07%, Kidney PC 1.68%). This suggests that TLR4^+^CXCR4^+^ PCs are an important pathogenic plasma cell subset in lupus nephropathy, which is consistent with a report by Ma et al*.* in which they demonstrated the significance of TLR4^+^CXCR4^+^ PCs for autoantibody production and glomerulonephritis development in LN [34]. In another study of pathogenic PC in the development of LN, they found that the frequency of TLR4 ^+^ CXCR4 ^+^ PCs was positively correlated with the production of dsDNA antibodies. After blocking TLR4, the secretion of dsDNA and other autoantibodies was significantly reduced, and the kidney damage was also improved [34]. In addition, the terminal differentiation of B cells requires participation of the Nrf2-regulated oxidative stress response. In fact, hydrogen peroxide (H_2_O_2_) promotes B cell proliferation and differentiation [38]. Our study suggests that Nrf2 gene overexpression (Blood PCs 0.28%, Blood PC 0.92%; Kidney PCs 5.75%, Kidney PC 2.06%) regulates TLR4^+^CXCR4^+^ PCs and PC levels in the blood and kidneys of a mouse model of lupus, with concomitant amelioration of damaged tissue. These results suggest that TLR4^+^CXCR4^+^ PCs may play an important role in autoantibody production in active LN and that the lack of TLR4 affects cytokine production related to LN development.

Oxidative stress imbalance is involved in LN pathogenesis. Furthermore, Nrf2 is a key regulator of the antioxidant stress pathway [39, 40]. Under oxidative stress, the increased electrophilic reagent or ROS reacts with Keap1 cysteine residues, resulting in structural changes. This prevents ubiquitination of Nrf2, which accumulates in the cytoplasm and is transferred to the nucleus, where it binds to small musculoaponeurotic fibrosarcoma (sMaf). The Nrf2-sMaf dimer can recognize ARE in the nucleus and induce the transcription of antioxidant factors, such as glutathione S-transferase Alpha 2, NAD(P)H: quinone oxidoreductase 1, glutamate cysteine ligase, and heme oxygenase-1 (HO-1) [41–44].

Here, we show decreased Nrf2 levels in MRL/lpr mice and increased levels of HMGB1/TLR4/NF-κB and its downstream inflammatory factors TNF-α and IL-1β. Nrf2 overexpression in MRL/lpr mice increased Nrf2 levels and decreased protein and cytokine levels in the corresponding inflammatory pathway. A study using dietary glycerol oleate to reduce LN also showed decreased Nrf2 levels in the kidneys of LN mice, which increased after the administration of dietary glycerol oleate and its derivatives. Subsequently, this inhibited the expression of pro-inflammatory biomarkers such as IL-1β and iNOS. These authors suggested that nephritis amelioration is associated with activation of the Nrf2 antioxidant stress pathway [45]. MRL/lpr mice developed more severe glomerulonephritis after exposure to the environmental toxin bisphenol A due to decreased Nrf2 expression [46]. Thus, Nrf2 may play an important regulatory role in preventing LN progression. However, Morito et al*.* showed that Nrf2 deficiency prolonged the lifespan of female lupus mice and speculated that Nrf2 deficiency enhanced apoptosis, which coincidentally improved the abnormal apoptosis feature of this mouse model. Additionally, the level of Nrf2 was related to lupus mouse strains and the role of Nrf2 in different stages of the disease [47, 48]. Nonetheless, the specific mechanisms underlying these observations require further investigation. In patients with LN, Nrf2 expression usually fluctuates slightly at the RNA level but markedly at the protein level [49]. As LN remains active for a long period with high oxidative stress, the antioxidant capacity of the body is gradually consumed, and Nrf2 content decreases.

Extracellular HMGB1 has been reported to increase macrophage inflammation and is highly correlated with histopathological features of renal injury in active nephritis via the TLR4/MyD88/NF-κB/p65 signaling pathway in LN [50–52]. TNF-α and IL-1β are expressed as NF-κB target genes that trigger inflammation, whereas TNF-α can stimulate the release of HMGB1 and amplify its inflammatory effect [25, 53].

Several studies have found that HMGB1, as a potential biomarker of cLN, is positively correlated with disease activity and negatively correlated with renal function [54, 55]. We have known that TLR can up-regulate cytokine levels and maintain inflammatory levels of LN. In a single-center cohort study, TLR2 and TLR4 were implicated in the pathogenesis of cLN [56]. Another study showed that TLR7 and TLR9 cause renal cell injury by regulating downstream NF-κB expression. Meanwhile, they suggesting that the TLR/NF-κB pathway is a potential therapeutic target for LN. Here, we showed increased levels of HMGB1, TLR4, NF-κB and their downstream target genes TNF-α and IL-1β in MRL/lpr mice, compared with control mice, as well as more severe inflammatory damage in kidney tissues.These results indicate the strong inflammation-mediating role of the HMGB1/TLR4/NF-κB pathway in LN. However, there are limited data on the pathogenesis of cLN. Based on the severity of the course, treatment and prognosis, we estimate that the inflammatory response caused by HMGB1/TLR4/NF-κB is more obvious in cLN.

Nrf2 inducers play important roles in alleviating LN inflammation and delaying disease progression [57–60]. One potential mechanism is Nrf2-induced alleviation of HMGB1-mediated oxidative damage [61, 62]. In our study, we successfully overexpressed Nrf2 in MRL/lpr mice with consequent inhibition of HMGB1 and the TLR4/NF-κB pathway, as well as its downstream inflammatory cytokines. This inhibitory mechanism may involve the Nrf2 target gene HO-1, which inhibits HMGB1 transfer to the cytoplasm, thereby alleviating inflammatory damage [63, 64]. Enhanced antioxidant capacity (regulated by Nrf2) can also inhibit ROS production, reducing oxidative stress and HMGB1 extracellular release [65, 66]. Thus, we speculate that Nrf2 inhibitory effect may occurs through its downstream antioxidant elements.

Currently, cortisol hormones, immunosuppressants (included mycophenolate mofetil and cyclophosphamide), anti-rheumatic drugs, and biologics (included belimumab and rituximab) are main treatment options for SLE and LN. However, such as the side effects of hormones and immunosuppressants for a long-term use and the limited safety and efficacy evidence for biologics in cSLE that affect the treatment efficacy and outcome [67–69]. The high morbidity and mortality of cLN requires us to delve deeper into the pathogenesis of LN and discover potential therapeutic targets. As our study demonstrated that the potential role of Nrf2/HMGB1/TLR4/NF-κB pathway in LN. The treatment of refractory LN with Nrf2, HMGB1 agonists has shown potential in several studies [70–72].

Unfortunately, there are some shortcomings in our study. Firstly, this study was conducted in a relatively small number of animal populations, so it cannot fully represent the SLE and LN populations. Second, although MRL/lpr mice are commonly used as model mice for studying SLE, it needs to be further confirmed whether they can be fully equivalent to human SLE due to racial limitations. We still need larger sample sizes and animal models that are closer human SLE for more accurate and effective studies.

## Conclusion

Our study showed that TLR4^+^CXCR4^+^ PCs increased significantly in MRL/lpr mice with severe renal injury. Furthermore, the HMGB1/TLR4/NF- κB pathway plays an important role in LN development. Nrf2, an antioxidant stress regulator, inhibits this pathway. Therefore, the Nrf2/HMGB1/TLR4/NF-κB pathway plays an important regulatory role in the development and progression of LN. We also highlighted the dual role of TLR4 in B-lymphocyte proliferation, differentiation, and the inflammatory pathway mentioned above. TLR4^+^CXCR4^+^ PCs, Nrf2, and HMGB1 might have therapeutic significance in LN and therefore could serve as effective targets in future therapeutic strategies.

## Data Availability

The data used to support the findings of this study are available from the corresponding author upon request.

## Abbreviations

SLE: Systemic lupus erythematosus
LN: Lupus nephritis
cSLE: Childhood-onset SLE
cLN: Childhood-onset LN
Nrf2: Nuclear factor erythroid-derived 2-related factor 2
Keap1: Kelch-like ECH-associated protein
HMGB1: High mobility group protein box 1
DAMP: Damage-Associated Molecular Pattern
RAGE: Receptors for advanced glycation end products
TLR2: Toll-like receptor 2
TLR4: Toll-like receptor 4
INF: Interferon
NF-κB: Nuclear Factor kappa B
sMaf: Small musculoaponeurotic fibrosarcoma
TNF-α: Tumor necrosis factor-alpha
IL-1β: Interleukin-1 beta
PC: Plasma Cells
PCs: Plasma Cells subset
SPF: Specific pathogen free
HE: Hematoxylin–eosin
ELISA: Enzyme-linked immunosorbent assay
qPCR: Quantitative polymerase chain reaction
WB: Western blotting
ROS: Reactive oxygen species
Neh: Nrf2-ECH homologous domains
HO-1: Heme oxygenase-1
